# The FABD domain is critical for the oncogenicity of BCR/ABL in chronic myeloid leukaemia

**DOI:** 10.1186/s12964-024-01694-8

**Published:** 2024-06-07

**Authors:** Renren Zheng, Wei Wei, Suotian Liu, Dachuan Zeng, Zesong Yang, Jie Tang, Jinfeng Tan, Zhenglan Huang, Miao Gao

**Affiliations:** 1 Department of Clinical Hematology, Key Laboratory of Laboratory Medical Diagnostics Designated by Ministry of Education, School of Laboratory Medicine, Chongqing Medical University, Chongqing, China; 2 Department of Clinical Laboratory, Women and Children’s Hospital of Chongqing Medical University, Chongqing, China; 3 Department of Clinical Laboratory, Chongqing Health Center for Women and Children, Chongqing, China; 4 Department of Hematology, The First Affiliated Hospital of Chongqing Medical University, Chongqing, China; 5 Department of Laboratory Medicine, The First Affiliated Hospital of Chongqing Medical University, Chongqing, China

**Keywords:** F-actin binding domain, BCR/ABL, Chronic myeloid leukaemia, Oncogenicity, p73

## Abstract

**Background:**

Abnormally expressed BCR/ABL protein serves as the basis for the development of chronic myeloid leukaemia (CML). The F-actin binding domain (FABD), which is a crucial region of the BCR/ABL fusion protein, is also located at the carboxyl end of the c-ABL protein and regulates the kinase activity of c-ABL. However, the precise function of this domain in BCR/ABL remains uncertain.

**Methods:**

The FABD-deficient adenovirus vectors Ad-BCR/ABL△FABD, wild-type Ad-BCR/ABL and the control vector Adtrack were constructed, and 32D cells were infected with these adenoviruses separately. The effects of FABD deletion on the proliferation and apoptosis of 32D cells were evaluated by a CCK-8 assay, colony formation assay, flow cytometry and DAPI staining. The levels of phosphorylated BCR/ABL, p73, and their downstream signalling molecules were detected by western blot. The intracellular localization and interaction of BCR/ABL with the cytoskeleton-related protein F-actin were identified by immunofluorescence and co-IP. The effect of FABD deletion on BCR/ABL carcinogenesis in vivo was explored in CML-like mouse models. The degree of leukaemic cell infiltration was observed by Wright‒Giemsa staining and haematoxylin and eosin (HE) staining.

**Results:**

We report that the loss of FABD weakened the proliferation-promoting ability of BCR/ABL, accompanied by the downregulation of BCR/ABL downstream signals. Moreover, the deletion of FABD resulted in a change in the localization of BCR/ABL from the cytoplasm to the nucleus, accompanied by an increase in cell apoptosis due to the upregulation of p73 and its downstream proapoptotic factors. Furthermore, we discovered that the absence of FABD alleviated leukaemic cell infiltration induced by BCR/ABL in mice.

**Conclusions:**

These findings reveal that the deletion of FABD diminished the carcinogenic potential of BCR/ABL both in vitro and in vivo. This study provides further insight into the function of the FABD domain in BCR/ABL.

## Introduction

Chronic myeloid leukaemia (CML) is a common haematological malignancy in adults [1]. The BCR/ABL oncoprotein is the basis for the development and progression of CML [2]. It is encoded by the bcr/abl fusion gene, which is generated from the translocation of the c-abl gene on chromosome 9 to the bcr gene on chromosome 22 [3–5]. The tyrosine kinase domain (SH1) of BCR/ABL is important for the pathogenicity of BCR/ABL. ATP binds to the ATP binding site of SH1, thereby constitutively activating the BCR/ABL tyrosine kinase [6, 7]. In addition, the parental tyrosine kinase c-ABL is located in the nucleus, while the BCR/ABL fusion protein is located in the cytoplasm [8]. Consequently, the tyrosine kinase activity of BCR/ABL is no longer strictly regulated by the nucleus, resulting in the activation of a variety of survival signals, including Ras/MAPK, Jak/Stat, and PI3K/Akt [9, 10], which, in turn, promotes the development and progression of CML [11, 12].

Imatinib, which is designed to disrupt the binding of ATP to the ATP-binding domain of BCR/ABL, has become the first-line treatment for CML. The application of imatinib has achieved great progress in clinical practice. However, resistance and intolerance to imatinib hinder effective CML treatment [13–15]. Therefore, the development of alternative inhibitors and strategies is required to maintain disease remission [16–18].

**Fig. 1:**
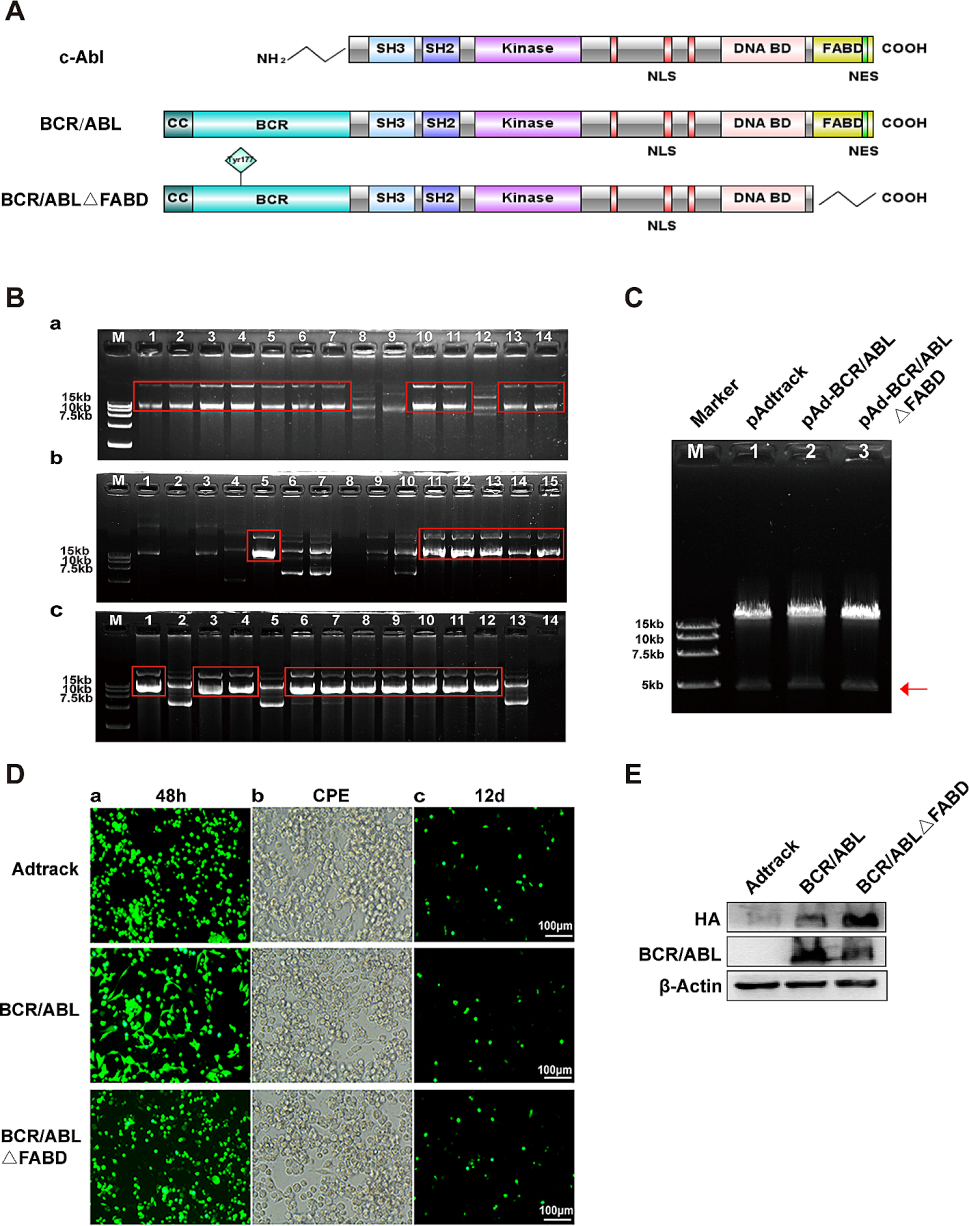
The expression of the BCR/ABL and BCR/ABLΔFABD proteins was successfully achieved using adenoviral vectors. (**A**) Schematic diagrams of c-ABL, BCR/ABL and BCR/ABLΔFABD. (**B**) The shuttle plasmids pAdtrack-CMV (a), pAdtrack-BCR/ABL (b) and pAdtrack-BCR/ABLΔFABD (c) were recombined with p-AdEasy-1 in BJ5183 bacteria. Agarose gel electrophoresis was then performed to screen the proper recombinant plasmids. The successful recombinant plasmids exhibited a greater molecular weight. The red rectangles in each agarose gel highlight typical successful recombinant plasmids. (**C**) Identification of recombinant adenoviral plasmids by Pac I enzyme digestion. M represents the DL15000 DNA marker; Lanes 1, 2 and 3 represent the recombinant adenoviral plasmids pAdtrack, pAd-BCR/ABL and pAd-BCR/ABL△FABD digested by PacI respectively. The correct recombinant adenovirus plasmids could be digested by the PacI restriction endonuclease to liberate both inverted terminal repeats (LITR and RITR), resulting in the release of a larger 30 kb fragment and a smaller 4.5 kb or 3 kb fragment (marked with a red arrow) [28]. (**D**) Packaging of adenoviruses in AD293 cells. AD293 cells were transfected with the respective recombinant adenoviral plasmids. (a) Then, the fluorescence intensity was detected at 48 h after transfection. (b) Cytopathic effects (CPEs) were observed under an inverted microscope 12 days later. (c) The fluorescence intensity decreased after 12 days of transfection. (**E**) 32D cells were infected with Ad-Adtrack, Ad-BCR/ABL, or Ad-BCR/ABLΔFABD adenoviruses for 48 h. The expression of BCR/ABL or BCR/ABLΔFABD was then detected by western blot analysis with an HA antibody or a c-ABL antibody. The HA tag was incorporated into the N-terminal region of BCR/ABL and BCR/ABLΔFABD

It has been reported that BCR/ABL remains active in imatinib-resistant CML cells, which means that not only the kinase domain but also other domains play essential roles in the function of the BCR/ABL oncoprotein. The F-actin binding domain (FABD) is located at the carboxyl end of both the BCR/ABL and c-ABL proteins (Fig. 1A) and mediates the binding of c-ABL with F-actin, resulting in feedback inhibition of c-ABL kinase activity [19]. The deletion of the amino acids that mediate the binding of c-ABL with F-actin in the FABD can restore the kinase activity of c-ABL [20, 21]. Moreover, a localization determinant is found in the last exon in the C-terminal region of c-ABL, which includes three nuclear localization signals (NLSs) [22–25] and a nuclear export signal (NES) contained in the FABD sequence. Given the vital role of FABD in the function of c-ABL and the same structure of FABD in BCR/ABL and c-ABL, it is reasonable to hypothesize that FABD plays a crucial role in the pathogenicity of BCR/ABL [26, 27]. However, the precise function of this domain in BCR/ABL remains uncertain. Therefore, we explored the influence of FABD on the malignant phenotype of cells induced by the BCR/ABL oncoprotein and the related mechanisms. The data presented here suggest that FABD is a key factor in modulating the subcellular location of BCR/ABL and in diminishing the oncogenicity of BCR/ABL by promoting the apoptotic pathway, which is mediated by p73 and its downstream signals.

## Materials and methods

### Cell culture

32D cells were cultured and maintained in RPMI 1640 medium (Gibco, USA) supplemented with 10% foetal bovine serum (FBS) (Gibco, USA) and 0.01% IL-3 (R&D, USA). The 293T and AD293 cell lines were maintained in Dulbecco’s modified Eagle’s medium (Gibco, USA) supplemented with 10% FBS. 32D cells infected with BCR/ABL adenoviruses were maintained in complete RPMI 1640 medium without IL-3. The cells were cultured in a humidified atmosphere with 5% CO_2_ at 37 °C.

### Adenovirus production

We used the AdEasy system to express BCR/ABL and BCR/ABL variants lacking the FABD domain (named BCR/ABL△FABD) according to the manufacturer’s protocol [28, 29]. Briefly, the coding sequence of bcr/abl or bcr/abl lacking FABD (named bcr/abl△FABD) was cloned and inserted into the adenoviral shuttle vector pAdTrack-CMV. After linearization with Pme I (NEB, USA), the shuttle plasmid was recombined with pAdEasy-1 (the adenovirus backbone vector) in BJ5183 bacteria. The positive replication-deficient recombinants were linearized with Pac I and transfected into AD293 cells with Lipo2000 to generate complete adenoviruses. AD293 cells were harvested, and the primary adenoviruses were collected after three freeze‒thaw cycles 14 days later. Virus amplification and purification were carried out as previously described [29].

### Cell viability assay

A Cell Counting Kit-8 (CCK-8) assay was performed to assess cell viability. A total of 5 × 10^3^ cells were plated into each well of a 96-well plate in 100 µl of media and then infected with adenovirus for 24, 48, or 72 h. Ten microlitres of CCK-8 solution (Topscience, China) was added to each well at the indicated times, and the plates were incubated for another 3 h at 37 °C. The absorbance was detected with a 96-well plate reader at 450 nm. Each group was analysed in triplicate.

### Cell colony formation assay

One hundred cells subjected to different treatments were seeded into 96-well plates supplemented with semisolid medium and cultured at 37 °C for 10 days, after which the number of colonies in each group was counted. Each group was tested in quadruplicate, and this experiment was repeated three times.

### Kinase activity assay

32D cells were plated in dishes at a density of 2 × 10^6^ cells/dish. We designed blank group, BCR/ABL group, BCR/ABL + IM group, BCR/ABL-ΔFABD group and BCR/ABL-ΔFABD + IM group for this experiment. The cells in each group were infected with the corresponding adenoviruses for 48 h. Then, the cells were collected, and total cell protein was extracted. First, the solution was prepared according to the instructions, and a standard curve was prepared. Then, protein samples were added to prepare the reaction system. The relative fluorescence intensity (RFU) was detected by a standard instrument. The fluorescence density at a wavelength of λex540/λex590 nm was determined. The amount of ADP produced in each treatment group was calculated according to the standard curve to reflect the kinase activity.

### Western blot analysis

Cells were collected at 48–72 h after adenovirus infection and lysed in RIPA buffer (CST, USA) supplemented with phosphatase and protease inhibitors. Equal amounts of each extract were separated by 8–10% sodium dodecyl sulfate‒polyacrylamide gel electrophoresis (SDS‒PAGE) and transferred onto polyvinylidene fluoride (PVDF) membranes (Millipore, Boston, MA). The indicated proteins were incubated with specific primary antibodies at 4 °C overnight and then incubated with the corresponding horseradish peroxidase-conjugated secondary antibodies at room temperature for 2 h. The blot was developed with super ECL reagent (Baoguang, China).

### Coimmunoprecipitation assay

Cells were collected at 48–72 h after adenovirus infection, washed with ice-cold PBS, and then lysed in immunoprecipitation buffer (Beyotime, China) for protein extraction. Protein A/G magnetic beads (MCE, USA) were incubated with an anti-BCR/ABL antibody (Santa, USA) on a shaker for 2 h. The beads were washed and then incubated with protein extracted from adenovirus-infected cells overnight on a shaker at 4 °C. The next day, immunoblot analysis was performed to identify the proteins.

### Immunofluorescence assay

The cells were collected and smeared onto slides. The cells were fixed with 4% paraformaldehyde, permeabilized with 0.1% Triton X-100, blocked with goat serum, and then incubated with primary antibodies (1:100 diluted in 5% goat serum) overnight at 4 °C. After washing, the cells were incubated with Cy3-conjugated secondary antibodies (Invitrogen, USA) for 1 h in the dark. Afterwards, the cell nucleus was stained with 4,6-diamidino-2-phenylindole. Finally, the fluorescence in the cells was observed with a confocal microscope.

### Murine leukaemia model

BALB/c mice (5–6 weeks old) were purchased from the Laboratory Animal Center of Chongqing Medical University and kept in the IVC for 1–2 weeks before use. Fifteen mice were randomly assigned to 3 groups: (1) the BCR/ABL group, which was injected with 32D cells infected with Ad-BCR/ABL, (2) the BCR/ABL△FABD group, which was injected with 32D cells infected with Ad-BCR/ABL△FABD, and (3) the blank group, which was injected with PBS. A total of 5 × 10^6^ cells were injected into each mouse through the tail vein. The state of the mice was monitored from the first day of injection until death. The mice were euthanized when they became moribund. All the mice were sacrificed after 3 months. The spleen, liver and lung were removed, and tissue sections were prepared and stained with haematoxylin and eosin (HE). Peripheral blood cells and bone marrow cells were collected for Wright‒Giemsa staining. All animal experiments were approved by the Institutional Animal Care and Use Committee of Chongqing Medical University.

### Statistical analysis

Statistical analysis was performed with GraphPad Prism 8.0 software. All the results are presented as the means ± SDs. A t test was used for comparisons between two groups, and one-way ANOVA was used to assess the significance of differences among three or more groups. *P* < 0.05 was considered to indicate a statistically significant difference.

## Results

### The BCR/ABL and BCR/ABLΔFABD proteins were successfully expressed with adenoviral vectors

A structural diagram of the BCR/ABL and BCR/ABLΔFABD proteins is shown in Fig. 1A. First, we constructed the FABD-deficient adenoviral vector Ad-BCR/ABLΔFABD, the wild-type BCR/ABL adenoviral vector Ad-BCR/ABL and the control vector Adtrack. The corresponding sequence of the bcr/abl gene or bcr/ablΔFABD gene was then cloned and inserted into the shuttle plasmid. Following digestion with PmeI, the shuttle plasmid was recombined with pAdEasy-1. The recombinant vectors that were successfully generated exhibited a higher molecular weight (Fig. 1B). The correct recombinant plasmids typically produce a larger 30 kb fragment and a smaller 4.5 kb or 3 kb fragment after being digested by Pac I (Fig. 1C). After linearization with Pac I, the recombinant plasmid was transfected into AD293 cells to produce adenoviruses. Fluorescence was detected, and then, plaques or cytopathic effects (CPEs) were observed up to 12 days after transfection (Fig. 1D). Then, the cells were harvested to collect the adenoviruses. The TCID_50_ values of Ad-BCR/ABL, Ad-BCR/ABLΔFABD and Adtrack were 10^7.5^/ml, 10^8^/ml and 10^8.25^/ml, respectively. 32D cells were infected with Adtrack, Ad-BCR/ABL or Ad-BCR/ABLΔFABD, and the corresponding proteins were then detected by western blotting. The results demonstrated that the BCR/ABL and BCR/ABLΔFABD proteins were successfully expressed in 32D cells via adenoviruses (Fig. 1E). In conclusion, the expression of the BCR/ABL and BCR/ABLΔFABD proteins with adenoviruses has been successfully achieved, thus paving the way for further research into the function of the FABD domain in the BCR/ABL protein.

### Deletion of FABD attenuates the ability of BCR/ABL to promote cell proliferation

**Fig. 2:**
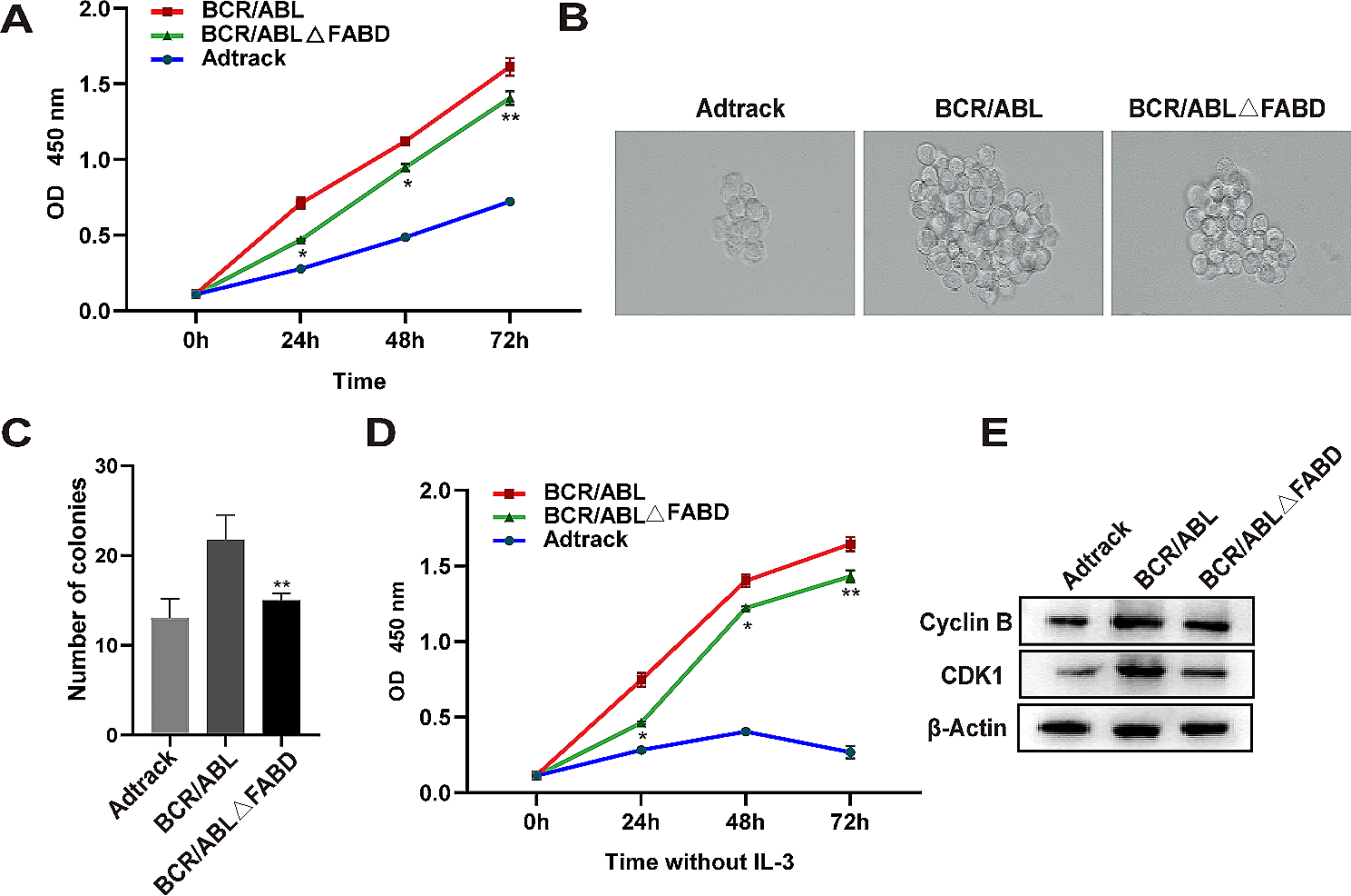
Deletion of FABD attenuates the ability of BCR/ABL to promote cell proliferation. (**A**) 32D cells were infected with Ad-Adtrack, Ad-BCR/ABL, or Ad-BCR/ABLΔFABD adenoviruses for the indicated times, after which cell viability was determined by a CCK-8 assay. (**B, C**) The effect of FABD deletion on the ability of cells to form colonies was assessed through a colony formation assay. 32D cells were infected with Ad-Adtrack, Ad-BCR/ABL, or Ad-BCR/ABLΔFABD adenoviruses for the indicated times, after which they were plated in semisolid medium. The number and morphology of the colonies were observed 10 days later. Each group was tested in quadruplicate, and the experiment was repeated three times. (**D**) The influence of FABD deletion on the transformation potential of BCR/ABL was evaluated through an IL-3 withdrawal experiment. 32D cells were infected with Ad-Adtrack, Ad-BCR/ABL, or Ad-BCR/ABLΔFABD adenoviruses and cultured in IL-3-free medium for the indicated times. Then, cell viability was determined by a CCK-8 assay. (**E**) 32D cells were infected with Ad-Adtrack, Ad-BCR/ABL, or Ad-BCR/ABLΔFABD adenoviruses for 48 h. The protein levels of Cyclin B and CDK1, which are associated with proliferation, were evaluated by western blotting

To assess the effect of FABD deletion on BCR/ABL-mediated cell proliferation promotion, CCK-8 and colony formation assays were carried out. The data from the CCK-8 assay indicated that FABD deletion could counteract the proliferation-promoting effect of BCR/ABL to a certain extent (Fig. 2A). Moreover, we found that compared with the wild-type (WT) BCR/ABL, the deletion of FABD resulted in a reduction in colony formation. The size and number of colonies were decreased by FABD deletion, as shown in Fig. 2B and C. In addition, a decrease in IL-3 dependence is a sign of successful transformation by the oncoprotein BCR/ABL in 32D cells [30]. The proliferation of 32D cells was significantly inhibited in the absence of IL-3 in the Adtrack group. However, the dependence of 32D cells on IL-3 was abolished by BCR/ABL. BCR/ABLΔFABD was also found to confer IL-3 independence to 32D cells, but with a lower proliferative efficiency than that observed in the BCR/ABL group (Fig. 2D). Subsequently, we examined the expression of Cyclin B and CDK1, which are associated with proliferation. The protein levels of Cyclin B and CDK1 were decreased in the BCR/ABLΔFABD group in comparison to those in the BCR/ABL group, while they were increased in comparison to those in the Adtrack group (Fig. 2E). These results indicated that FABD deletion counteracted the proliferation-promoting effect of BCR/ABL to a certain extent but did not fully abolish the transformation ability of BCR/ABL.

### Deletion of FABD reduces the ability of BCR/ABL to antagonize apoptosis

**Fig. 3:**
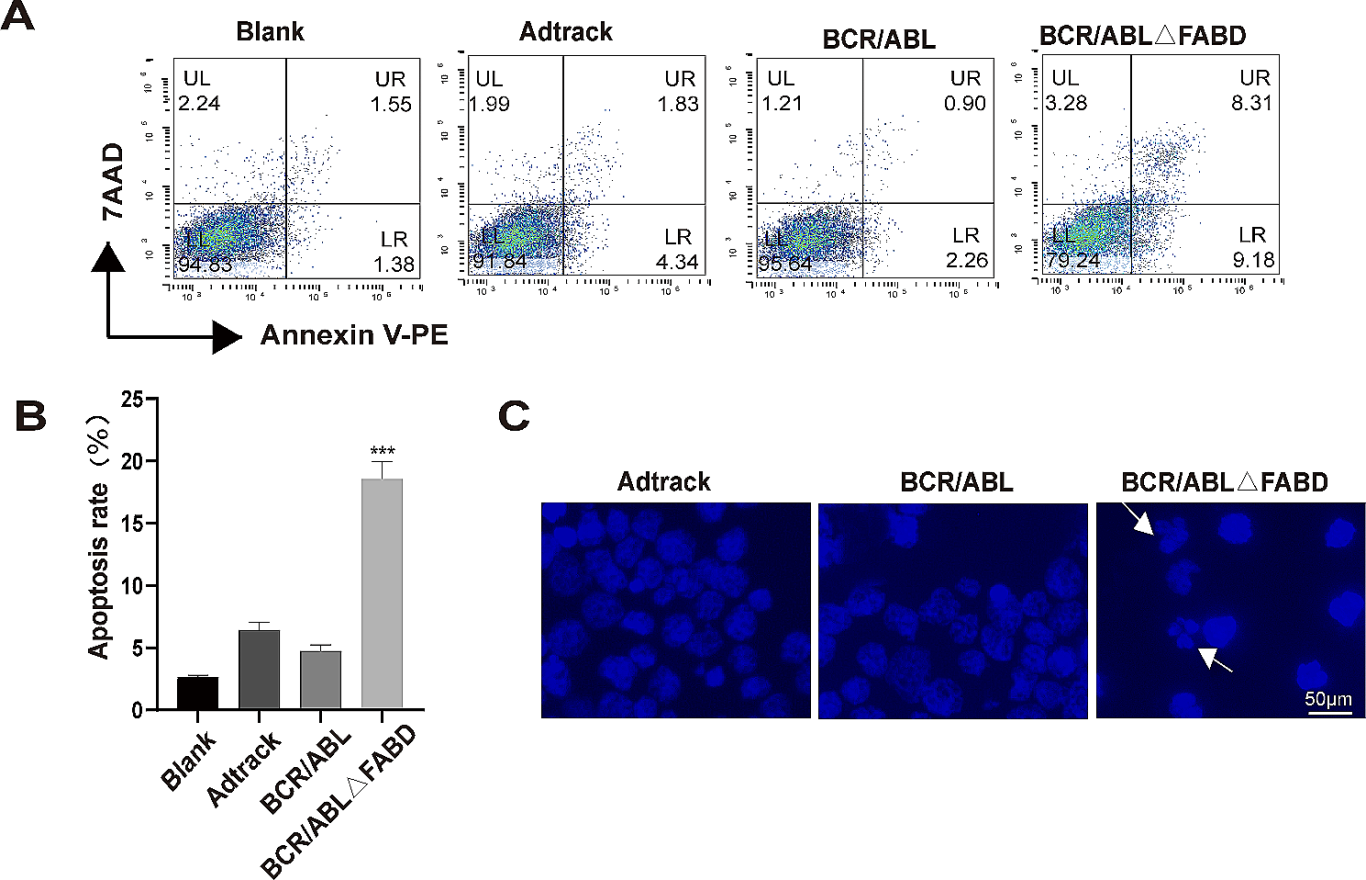
Deletion of FABD reduces the ability of BCR/ABL to antagonize apoptosis. (A) 32D cells were infected with Ad-Adtrack, Ad-BCR/ABL, or Ad-BCR/ABLΔFABD adenoviruses for a period of 48 h. Subsequently, apoptotic cells were identified by flow cytometry. The blank represents 32D cells with no treatment. Each experiment was conducted in triplicate. (**B**) Statistical analysis of the apoptosis results detected by flow cytometry. (**C**) 32D cells were infected with Ad-Adtrack, Ad-BCR/ABL, or Ad-BCR/ABLΔFABD adenoviruses for 48 h. Then, the nuclear morphology was observed following DAPI staining. The white arrow indicates typical nucleic fragmentation

To assess the effect of FABD on the antiapoptotic effect of BCR/ABL, we used the adenoviruses Ad-BCR/ABL and Ad-BCR/ABLΔFABD to infect 32D cells. Apoptotic cells were then detected by flow cytometry. The results showed that the proportion of apoptotic cells in the BCR/ABLΔFABD group was significantly greater than that in the BCR/ABL group (Fig. 3A **and B**). Furthermore, the results of DAPI staining demonstrated that a greater number of cells exhibiting typical apoptotic morphological characteristics, such as chromatin condensation and nucleic fragmentation, were observed in the BCR/ABLΔFABD group. No discernible alterations in nuclear morphology were observed in the BCR/ABL group (Fig. 3C), which was consistent with the flow cytometry results. In conclusion, the results of this section illustrated that the loss of FABD reduces the ability of BCR/ABL to antagonize apoptosis.

### Deletion of FABD relieves the severity of CML-like disease caused by BCR/ABL in mice

**Fig. 4:**
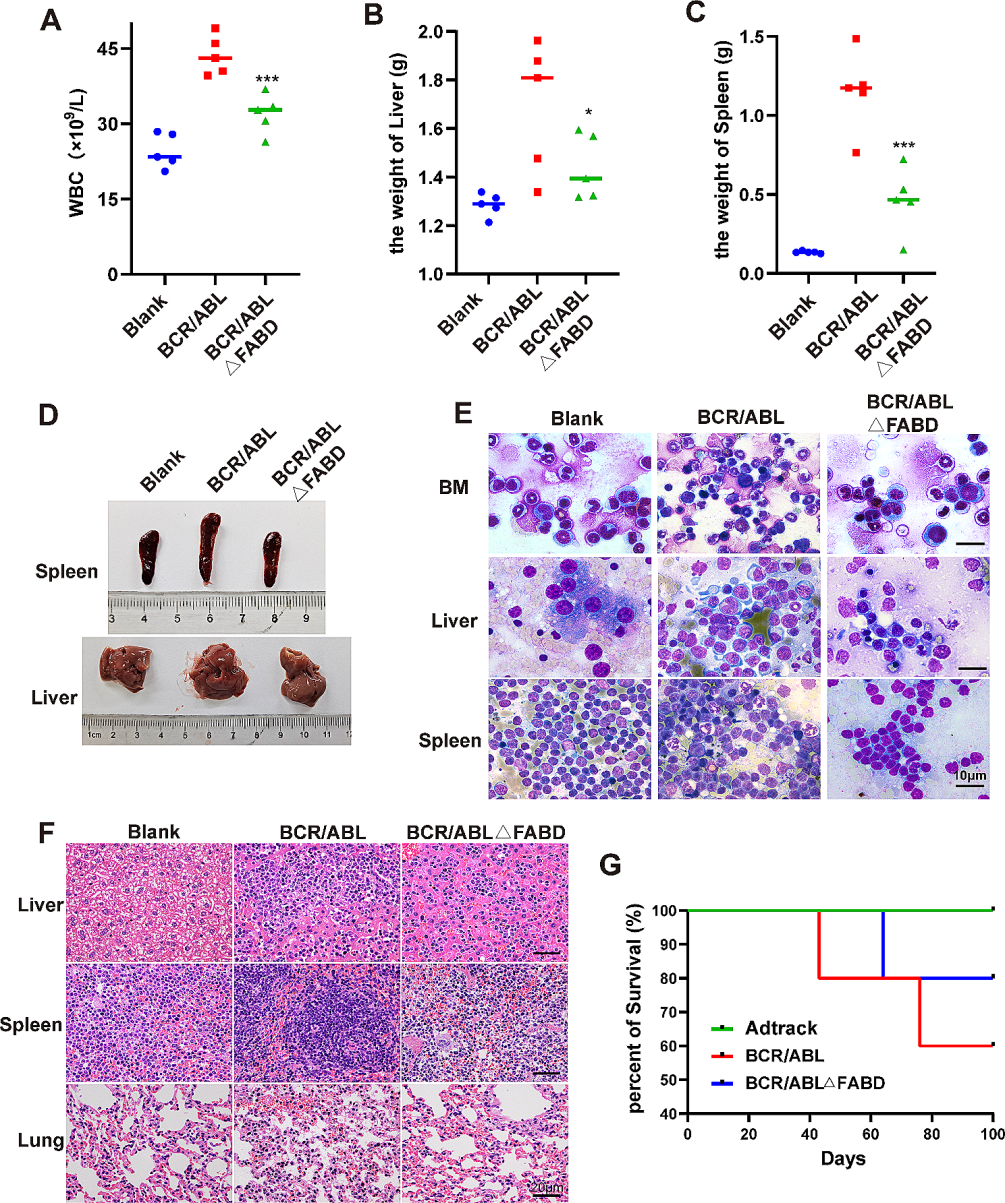
Deletion of FABD relieves the severity of CML-like disease caused by BCR/ABL in mice. A total of 5 × 10^6^ 32D cells infected with Ad-BCR/ABL or Ad-BCR/ABLΔFABD adenoviruses were injected intravenously into each BALB/c mouse. A control group of BALB/c mice was injected with PBS and designated the blank group. There were 5 mice in each group. (**A**) The number of white blood cells (WBCs) in the peripheral blood was counted and recorded every week. The peak value of the WBC in each mouse was recorded and statistically analysed. (**B, C**) The weights of the liver and spleen of each mouse were measured at the time of execution and statistically analysed. (**D**) Representative images of the spleens and livers of mice from each group were shown. (**E**) Cells from the bone marrow, liver, and spleen of mice were stained with Wright‒Giemsa stain. The infiltration of leukaemic cells in these tissues was observed after staining. (**F**) Tissues from mouse liver, spleen, and lung were sectioned and stained with HE. The infiltration of leukaemic cells in these tissues was observed after staining. (**G**) The survival status of each group was recorded and analysed. The survival curves were generated with GraphPad 8.0

To evaluate the influence of FABD deletion on the tumorigenesis of BCR/ABL in vivo, CML mouse models were established. 32D cells infected with the corresponding adenovirus were injected into BALB/c mice via the tail vein. A subset of mice developed typical CML-like disease between 5 and 8 weeks postinjection. The diseased mice exhibited weight loss, a significant increase in neutrophils in peripheral blood, and hepatosplenomegaly with myeloid cell infiltration and lung parenchymal infiltration. The results showed that the number of white blood cells (WBCs) in the BCR/ABLΔFABD group was lower than that in the BCR/ABL group but greater than that in the blank group (Fig. 4A**).** Additionally, the weights of the liver and spleen were lower in the BCR/ABLΔFABD group than those in the BCR/ABL group (Fig. 4B and C). Concomitantly, the BCR/ABL group exhibited more pronounced hepatosplenomegaly, with splenomegaly being particularly evident. (Fig. 4D). Cells from the bone marrow, liver, or spleen were stained with Wright‒Giemsa stain to examine the infiltration of leukaemic cells. Compared to the wild-type BCR/ABL group, the FABD deletion group exhibited significantly reduced leukocyte infiltration in the spleen and liver and a decreased number of nucleated cells in the bone marrow (Fig. 4E). Tissue sections from the liver, spleen, and lung were subjected to HE staining to evaluate the extent of leukaemic cell infiltration. The results showed that the infiltration of leukaemic cells in the liver and spleen was more pronounced in the diseased mice in the BCR/ABL group, while spontaneous lung metastasis and splenic metastasis with fewer invasive granulocytes were observed in the BCR/ABLΔFABD group (Fig. 4F). The observation period continued for 100 days, during which no significant difference in survival was observed between the wild-type BCR/ABL group and the FABD truncated mutant BCR/ABL group (Fig. 4G). However, the incidence of CML-like disease in the BCR/ABLΔFABD group was lower than that in the BCR/ABL group (20% vs. 40%). The time required for the first mouse to develop CML-like disease was obviously longer in the BCR/ABLΔFABD group than in the BCR/ABL group (41 vs. 62 days). In conclusion, the in vivo data suggested that FABD deletion relieved the severity of CML-like disease caused by BCR/ABL to a certain degree but did not impair the tumorigenesis of BCR/ABL.

### FABD deletion increases p-Y177 levels but does not rely on p-Y177 to function

**Fig. 5:**
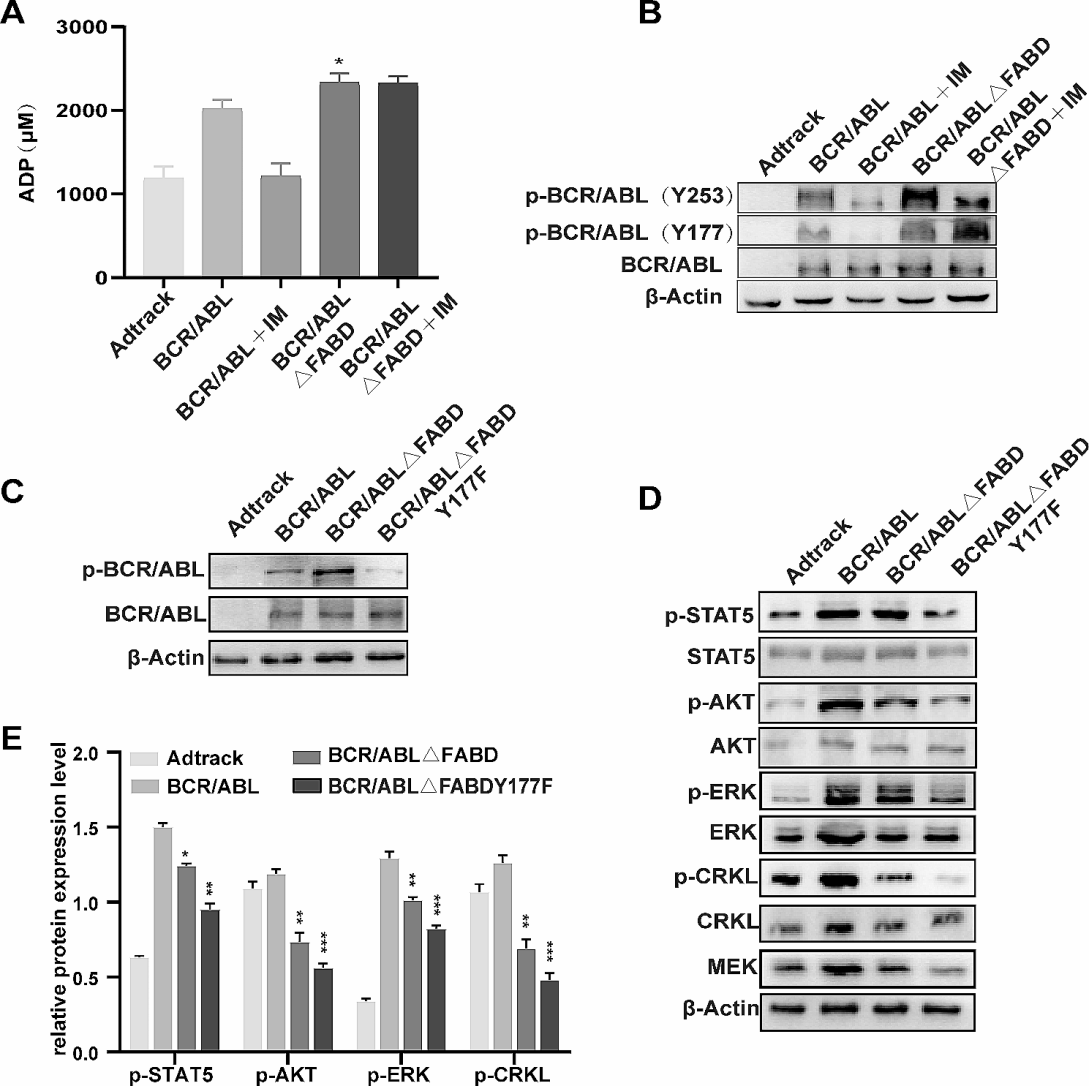
FABD deletion increases p-Y177 levels. (A) 32D cells were infected with Ad-Adtrack, Ad-BCR/ABL, Ad-BCR/ABL△FABD or Ad-BCR/ABLΔFABD-Y177F adenoviruses for the indicated times. Then, total protein kinase activity was tested by a kinase activity assay according to the kit instructions. (**B**) The levels of p-BCR/ABL (Y253) and p-BCR/ABL (Y177) in each group were evaluated by western blotting. (**C**) The tyrosine at the Y177 site in BCR/ABLΔFABD was mutated to phenylalanine. The expression of p-BCR/ABL or BCR/ABL was evaluated by western blotting after infection with the respective adenoviruses. (**D**) The expression of p-STAT5, p-AKT, p-ERK, p-CRKL and MEK was evaluated by western blotting after infection with the respective adenoviruses. (**E**) The ratio of p-STAT5, p-AKT, p-ERK or p-CRKL to the corresponding total protein was quantified

The data presented above allow us to conclude that FABD plays a role in the pathogenicity of BCR/ABL. However, the precise mechanism by which FABD affects BCR/ABL oncogenicity remains unknown. The constitutively activated kinase is a key factor in the tumorigenesis of BCR/ABL. It is of interest to ascertain whether FABD participates in the activation of BCR/ABL kinase. First, we detected the overall kinase activity of adenovirus-infected cells. We found that BCR/ABL increased overall kinase activity, and the deletion of FABD further increased overall kinase activity. The kinase activity of BCR/ABL was inhibited by imatinib, while the increase in kinase activity resulting from FABD deletion was not inhibited by imatinib (Fig. 5A). These data indicated that the increase in phosphorylation caused by FABD deletion may lie outside of the kinase domain of BCR/ABL. Y177 and Y253 are two tyrosine residues that play vital roles in the activity of BCR/ABL. Y177 is located within the BCR protein, and Y253 is located within the kinase domain of ABL [31, 32]. The results indicated that the deletion of the FABD could increase the phosphorylation of Y177 and Y253. The phosphorylation of Y253 was suppressed by imatinib in both the BCR/ABL and BCR/ABLΔFABD groups. The phosphorylation of Y177 was inhibited by imatinib in the BCR/ABL group but not in the BCR/ABLΔFABD group (Fig. 5B). Therefore, we speculated that FABD might suppress the phosphorylation of Y177. Upon the deletion of FABD, the suppression exerted by the FABD on Y177 was alleviated. To verify this hypothesis, we introduced a mutation of tyrosine to phenylalanine at the Y177 site in BCR/ABLΔFABD, resulting in the BCR/ABLΔFABD-Y177F construct. Then, we detected the expression of p-BCR/ABL and its downstream molecules. As shown in Fig. 5C, the expression of p-BCR/ABL decreased significantly in the BCR/ABLΔFABD-Y177F group. Moreover, the levels of p-STAT5, p-AKT, p-ERK and p-CRKL decreased significantly in the BCR/ABLΔFABD-Y177F group. Notably, the expression of p-BCR/ABL increased in the BCR/ABLΔFABD group, while the p-STAT5, p-AKT, p-ERK and p-CRKL levels decreased to varying degrees in this group (Fig. 5D, E). These results demonstrated that the BCR/ABLΔFABD-Y177F mutation successfully reversed the kinase activation resulting from FABD deletion.

**Fig. 6:**
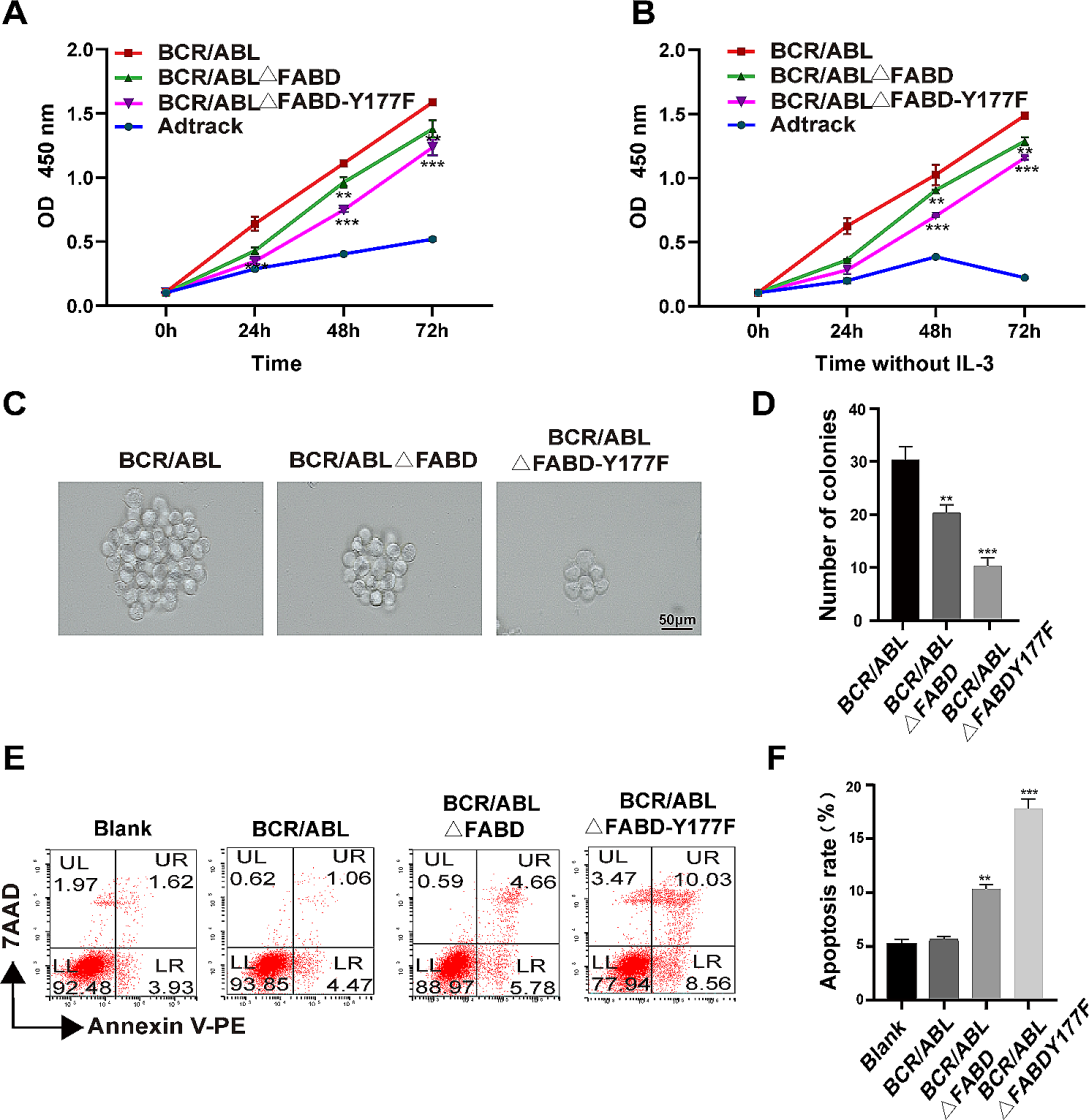
FABD deletion does not function through p-Y177 elevation. We detected whether Y177F was able to reverse the proliferation inhibition and apoptosis induction caused by BCR/ABLΔFABD. 32D cells were infected with Ad-Adtrack, Ad-BCR/ABL, Ad-BCR/ABL△FABD or Ad-BCR/ABLΔFABD-Y177F adenoviruses and then cultured in medium supplemented with (**A**) or without (**B**) IL-3 for the indicated times. The cell viability in each group was determined by a CCK-8 assay. For the colony-forming assay, 32D cells were infected with Ad-BCR/ABL, Ad-BCR/ABLΔFABD, or Ad-BCR/ABLΔFABD-Y177F adenoviruses for the indicated times and then plated in semisolid medium. The morphology (**C**) and number (**D**) of colonies were observed 10 days later. Each group was repeated three times. For the apoptosis detection assay, 32D cells were treated as described above, and apoptosis was examined by flow cytometry (**E).** The results for each group were statistically analysed (**F**)

Subsequently, we investigated the ability of the Y177F mutation to reverse the proliferation inhibition and apoptosis induction caused by BCR/ABLΔFABD. Compared to the BCR/ABL and BCR/ABLΔFABD groups, the BCR/ABLΔFABD-Y177F group exhibited a reduction in cell proliferation. (Fig. 6A). The number and the size of the colonies decreased in the BCR/ABLΔFABD-Y177F group (Fig. 6C, D). BCR/ABLΔFABD-Y177F was observed to hinder IL-3 independence in 32D cells, with a lower growth rate than that of BCR/ABL and BCR/ABLΔFABD. (Fig. 6B). The number of apoptotic cells was greater in the BCR/ABLΔFABD-Y177F group (Fig. 6E, F). In conclusion, the Y177F mutation was unable to reverse the apoptosis induction and inhibition of cell proliferation caused by FABD deletion. Interestingly, the BCR/ABLΔFABD-Y177F mutant further inhibited cell proliferation and induced apoptosis. This phenomenon might be related to the blockade of the Ras-MAPK signalling pathway by the Y177F mutation [33], as evidenced by the decrease in MEK and p-ERK levels (Fig. 5D, E). In conclusion, FABD deletion increased p-Y177 level in a manner that was independent of the BCR/ABL kinase. However, the increase in p-Y177 was not the mechanism by which FABD affects BCR/ABL oncogenicity.

**FABD deletion mediates the translocation of BCR/ABL to the nucleus, where it activates p73 to induce apoptosis**.

**Fig. 7:**
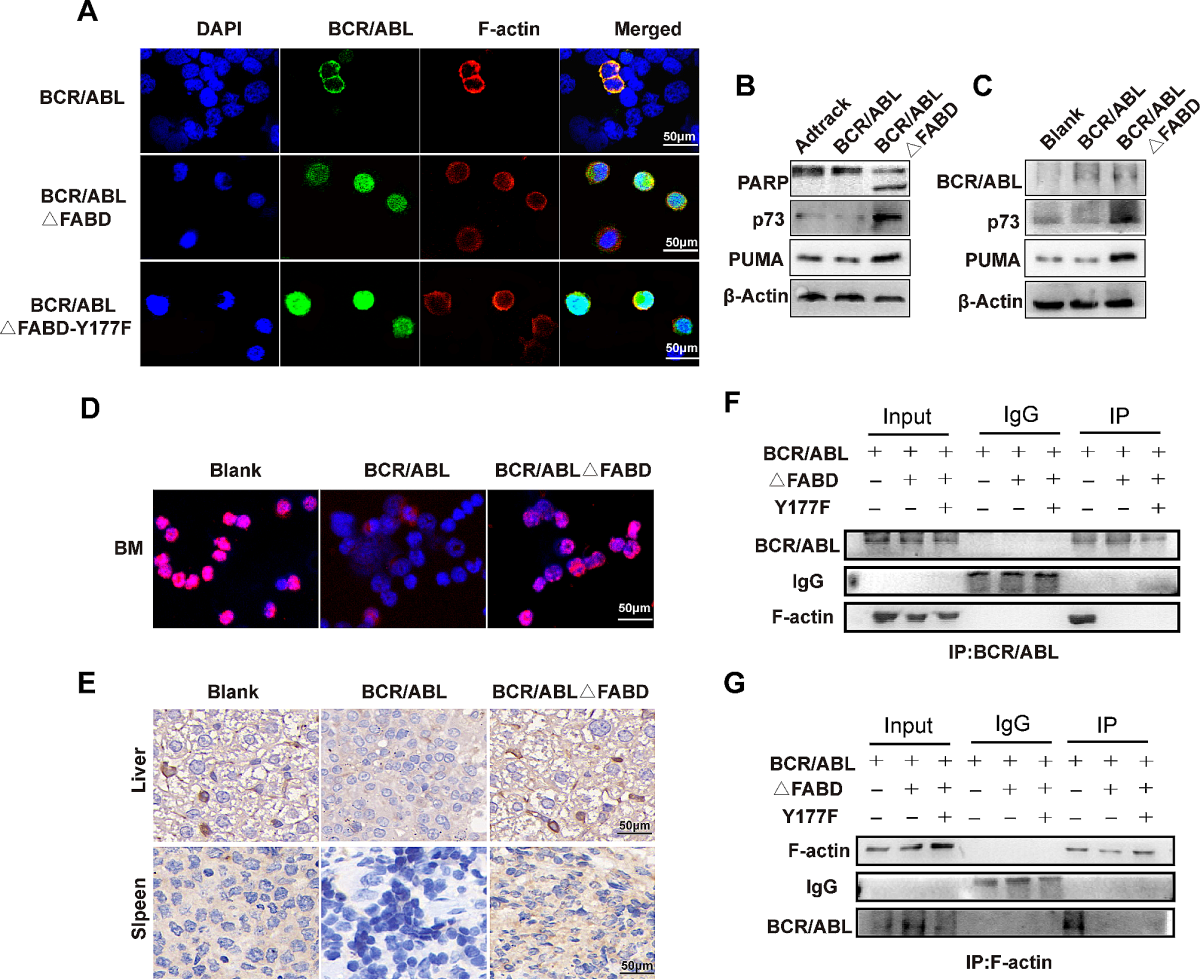
FABD deletion mediates the translocation of BCR/ABL into the nucleus, where it activates p73 to induce apoptosis. (**A**) 32D cells were infected with Ad-BCR/ABL, Ad-BCR/ABLΔFABD, or Ad-BCR/ABLΔFABD-Y177F adenoviruses for 48 h. Thereafter, the locations of BCR/ABL and F-actin were examined by immunofluorescence. The cell nuclei were stained with DAPI. BCR/ABL was visualized by a FITC-conjugated specific antibody, while F-actin was detected by a Cy-3-conjugated specific antibody. The scale represents 50 μm. (**B**) 32D cells were infected with the corresponding adenoviruses for 48 h, after which the expression of PARP, p73 and PUMA was analysed by western blotting. (**C**) The expression of BCR/ABL, p73 and PUMA in mouse bone marrow cells from the corresponding groups was analysed by western blotting. (**D**) The localization and expression of p73 in bone marrow cells were detected by immunofluorescence. (**E**) The expression of p73 in mouse liver and spleen cells from the corresponding groups was detected by immunohistochemistry. (**F, G**) 32D cells were infected with the corresponding adenoviruses for 48 h, after which the interaction between BCR/ABL and F-actin was analysed by coimmunoprecipitation. ΔFABD represents BCR/ABLΔFABD, and Y177F represents BCR/ABLΔFABD-Y177F

The NES is located within the FABD domain. Upon deletion of the FABD, there are only three NLSs present in BCR/ABLΔFABD, with no NES. We analysed the cellular localization of BCR/ABL and its mutants by immunofluorescence. We observed that the BCR/ABL protein was distributed evenly in the cytoplasm. BCR/ABLΔFABD and BCR/ABLΔFABD-Y177F were located in the nucleus (Fig. 7A). Previous studies have shown that c-ABL shuttle between the cytoplasm and the nucleus. Upon stimulation by DNA damage, the c-ABL protein translocates from the cytoplasm to the nucleus, where it induces cell apoptosis by activating the p73 protein and its downstream molecules. To determine whether a similar effect occurs in BCR/ABLΔFABD upon entry into the nucleus, we detected the expression of the p73 protein and PUMA, a downstream molecule of p73 and a proapoptotic factor. We found that the expression of p73 and PUMA was elevated in the BCR/ABLΔFABD group, accompanied by the cleavage of the apoptosis-related protein PARP (Fig. 7B). Then, we detected the expression of BCR/ABL, p73 and PUMA in the mouse bone marrow. The expression of BCR/ABL in bone marrow cells once again confirmed the successful establishment of the mouse leukaemia model. The expression of p73 and PUMA was also observed to increase in bone marrow cells following the deletion of FABD. (Fig. 7C). Next, immunofluorescence (IF) and immunohistochemistry (IHC) were used to evaluate the expression of p73 in mouse tissues. The results showed that the expression of p73 was upregulated in the bone marrow, liver and spleen compared to that in the BCR/ABL group. (Fig. 7D **and E**). Then, we detected the colocalization and interaction between BCR/ABL (or its mutant) and F-actin by immunofluorescence and coimmunoprecipitation assays. The results demonstrated that BCR/ABL was colocalized with F-actin. The colocalization disappeared in the BCR/ABLΔFABD and BCR/ABLΔFABD-Y177F groups (Fig. 7A). The interaction between BCR/ABL and F-actin was observed in the BCR/ABL group when the samples were immunoprecipitated with either the BCR/ABL antibody or the F-actin antibody. However, the interaction disappeared when FABD was deleted (Fig. 7F **and G**). In conclusion, the loss of FABD resulted in the translocation of BCR/ABL from the cytoplasm to the nucleus, which subsequently activated p73 and its downstream molecules, thereby promoting cell apoptosis. The translocation elicited by FABD deletion may be attributed to the disruption of the interaction between BCR/ABL and F-actin.

## Discussion

The key protein domains that regulate the kinase activity of BCR/ABL and mutations in drug-resistant cells have been specifically described [34]. However, the contribution of FABD to BCR/ABL carcinogenicity remains poorly understood. FABD, the functional domain of BCR/ABL for F-actin binding, is essential for high-affinity interplay with F-actin [35]. Therefore, it plays a crucial role in a variety of cellular behaviours, including cell adhesion, migration and cytoskeleton formation. It has been experimentally confirmed that based on the structure of F-actin, cells usually exhibit strong invasive features [36, 37]. In our study, compared to wild-type BCR/ABL, FABD-depleted BCR/ABL was less able to promote proliferation and inhibit apoptosis, suggesting that FABD may be involved in the tumorigenicity of BCR/ABL.

It has been reported previously that ABL kinase activity is essential for BCR/ABL leukaemogenesis [38, 39]. The c-ABL tyrosine kinase is inhibited by F-actin, and this inhibition can be relieved through the deletion of its FABD [20]. The results of this study were comparable to those of previous studies, indicating that the deletion of the FABD can increase the phosphorylation level of BCR/ABL. The BCR/ABLΔFABD-Y177F mutant could restore the increased phosphorylation levels caused by FABD deletion. Notably, despite the elevated kinase activity resulting from FABD deletion, FABD itself does not appear to function in this manner. Therefore, further research is required to elucidate the precise mechanism by which FABD regulates the malignancy of BCR/ABL.

A key consideration when evaluating the function of BCR/ABL is its location in the cell [40]. BCR/ABL has been found in the nucleus and interacts with its protein partner USP1 [41]. The subcellular localization of the protein in the nucleus or the cytoplasm depends on the balance between the nuclear localization signal (NLS) and nuclear export signal (NES) [25, 39, 40]. The effect of BCR/ABL C-terminal deletion on cell survival is a consequence of changes in the subcellular localization of the protein [39, 42]. In our study, the deletion of FABD with the loss of NES resulted in the blockage of a portion of BCR/ABL in the nucleus, leading to a reduction in the cytoplasmic functions of BCR/ABL. We have also shown that truncation of the C-terminus of BCR/ABL eliminates the association of BCR/ABL with actin myosin and microtubule filaments, thus enabling BCR/ABL to shuttle between the nucleus and cytoplasm. Therefore, BCR/ABL is more potential to enter into the nucleus and then be blocked in the nucleus. Our results also confirmed that these alterations could spatially inhibit the activation of survival signals, including the MAPK/ERK signalling pathway.

**Fig. 8:**
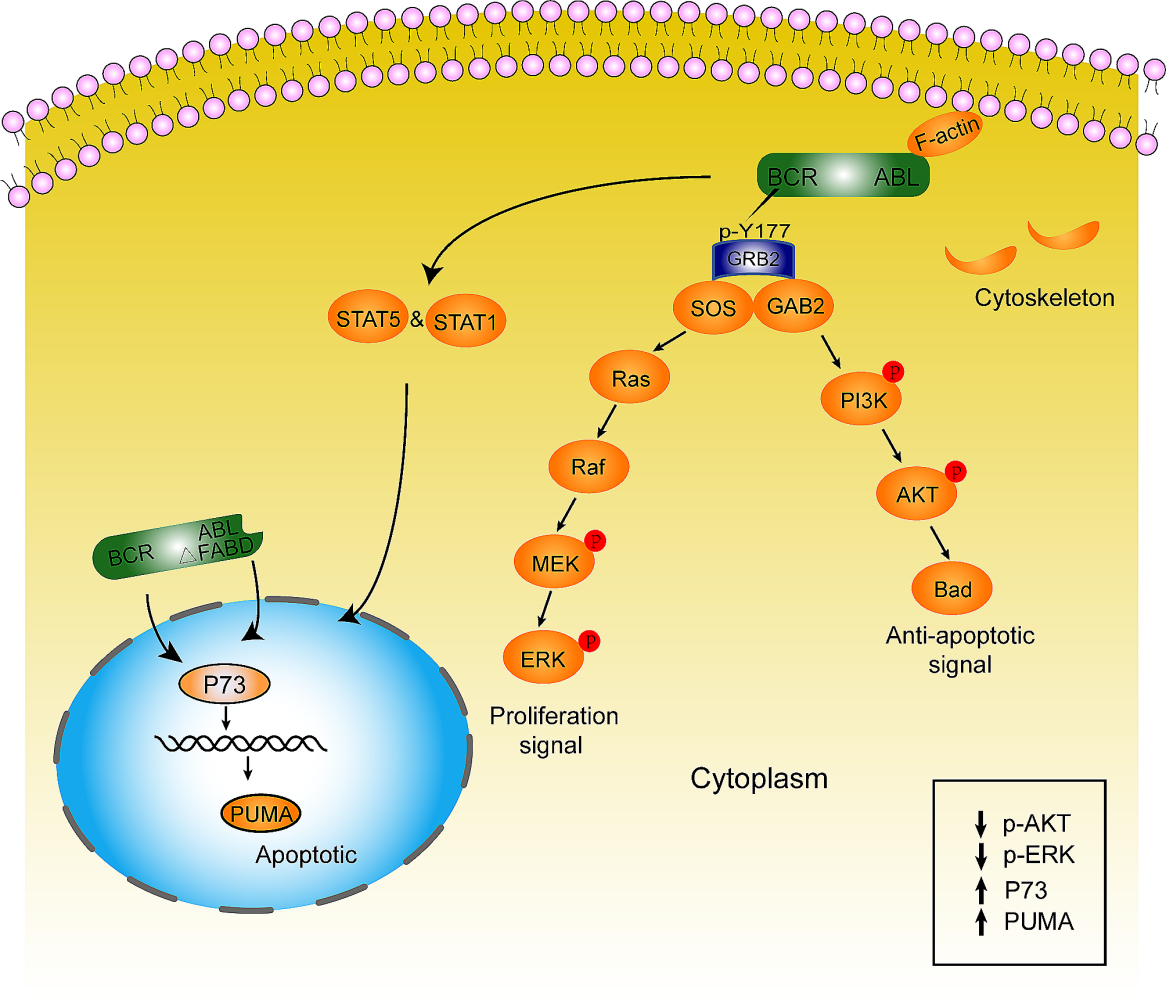
A schematic diagram of our study. Wild-type BCR/ABL is located in the cytoplasm through interactions with F-actin and stimulates downstream pathways to promote proliferation and antagonize apoptosis. When the FABD domain is deleted, the interaction between BCR/ABL and F-actin is interrupted. Then, BCR/ABLΔFABD translocates into the nucleus and stimulates p73 and its downstream molecules to induce apoptosis. Moreover, due to the decrease in cytoplasmic BCR/ABL, proliferative stimuli are weakened

More importantly, following the entry of BCR/ABL into the nucleus, it induces the expression of PUMA through the activation of p73 (Fig. 8). We found that the expression of PUMA, a downstream signalling molecule of p73, is increased after the loss of FABD, which in turn initiates Bax/Bcl-2-dependent programmed cell death [43, 44]. It has been reported that imatinib can also block BCR/ABL in the nucleus, which subsequently mediates cell apoptosis [39]. Moreover, in response to DNA damage agents, BCR/ABL translocates to the nucleus, leading to the interruption of normal DNA repair processes [45]. Furthermore, our findings indicate that FABD is important, albeit not fully efficient or sufficient, for BCR/ABL to induce CML-like disease in mice.

## Conclusions

In summary, FABD deletion has the potential to inhibit the ability of BCR/ABL to promote cell proliferation and to prevent apoptosis. The apoptosis induced by FABD deletion may be attributed to the translocation of BCR/ABL to the nucleus, where it subsequently activates the p73 pathway. Our research provides a more comprehensive understanding of the function of the FABD domain in BCR/ABL. Targeting this domain may facilitate the identification of novel therapeutic strategies to further impede the progression of chronic myeloid leukaemia.

## Data Availability

No datasets were generated or analysed during the current study.

## Abbreviations

Ad: Adenovirus
BM: Bone marrow
CML: Chronic myeloid leukaemia
FABD: F-actin binding domain
NES: Nuclear export signal
NLS: Nuclear localization signal
WBC: White blood cell
SH1: Src homology 1 domain
IL-3: Interleukin-3
IM: Imatinib
TCID50: Tissue culture infective dose
RFU: Relative fluorescence intensity
CPE: Cytopathic effect
